# A brief review on dengue molecular virology, diagnosis, treatment and prevalence in Pakistan

**DOI:** 10.1186/1479-0556-10-6

**Published:** 2012-08-28

**Authors:** Sobia Idrees, Usman A Ashfaq

**Affiliations:** 1 Department of Bioinformatics and Biotechnology, Government College University (GCU), Faisalabad, Pakistan

**Keywords:** Dengue virus, NS3 protease, Diagnosis, Medicinal plants, Prevalence

## Abstract

Dengue virus infection is a serious health problem infecting 2.5 billion people worldwide. Dengue is now endemic in more than 100 countries, including Pakistan. Each year hundreds of people get infected with dengue in Pakistan. Currently, there is no vaccine available for the prevention of Dengue virus infection due to four viral serotypes. Dengue infection can cause death of patients in its most severity, meanwhile many antiviral compounds are being tested against dengue virus infection to eradicate this disease but still there is a need to develop an efficient, low-cost and safe vaccine that can target all the four serotypes of dengue virus. This review summarizes dengue molecular virology, important drug targets, prevalence in Pakistan, diagnosis, treatment and medicinal plant inhibitors against dengue.

## Introduction

Dengue virus (DENV) infection is an important arthropod-born viral infection infecting about 2.5 billion people worldwide, of which approximately 975 million belong to large and small cities of tropical and sub-tropical countries in Southeast Asia, the Pacific and the America [1,2]. The prevalence of dengue has grown dramatically in recent decades and is now endemic in more than 100 countries [3]. Approximately, 50 to 100 million infections occur each year leading to 500,000 hospitalizations and 20,000 deaths, estimated by WHO [4]. Dengue virus was first isolated from Japan in 1942 by Hotta [5]. Dengue virus belongs to Flaviviridae family and is transmitted to humans by infective female of Aedes genus, especially *Aedes aegypti* or *Aedes albopictus* mosquito [6,7]. There are 4 related, but antigenically distinct serotypes of dengue virus (DEN 1–4) evolved from a common ancestor who manifest with similar symptoms [8,9]. Dengue virus causes two types of infections, primary infection and secondary infection. Primary infection results in acute febrile illness known as dengue fever (DF) which is cleared in approximately seven days by a complex immune response. Secondary infection is more severe and results in haemorrhagic fever (DHF) or dengue shock syndrome (DSS) [10]. Predominantly, it affects children in Southeast Asia and is characterized by increased vascular permeability, plasma leakage, haemorrhagic manifestations and thrombocytopenia. Both DHF and DSS can be fatal and can lead to death among the patients [11]. Pakistan due to its crowded cities, unsafe water, inadequate sanitation, large number of refugees and low vaccination coverage is at high risk of dengue endemics. [12]. To date, there is no vaccine available to combat dengue infection. The vaccine must be tetravalent to be effective in all four serotypes and there is no efficient animal model available for DHF/DSS. Therefore, developing vaccine against dengue is quite challenging. Dengue is a life-threatening fever that can cause death of patients in its most severity, currently many antiviral compounds are being tested against dengue virus infection to eradicate this disease but still there is a need to develop an efficient, low-cost and safe vaccine that can target all the four serotypes of dengue virus.

## DENV life cycle

**Figure 1:**
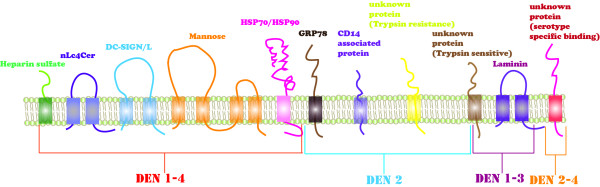
DENV cell entry receptors.

**Figure 2:**
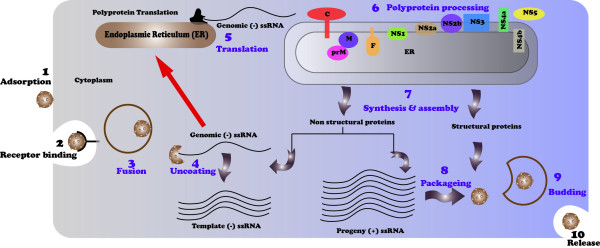
Dengue virus replication cycle.

To date, many research studies have been conducted to understand the life cycle of dengue virus, especially the viral protein processing and the genome replication. Dengue virus binds to its receptor, and this process is mediated by envelop protein (E). In mammalian cell, DEN 1–4 serotypes bind with Heparan sulfate, nLc4Cer, DC-SIGN/L-SIGN and Mannose receptors. DEN-2 serotype also binds with HSP70/HSP90, GRP78, CD14-associated protein and two unknown proteins having trypsin resistance and trypsin sensitive properties. DEN 1–3 serotypes as well bind with Laminin receptor. DEN 2–4 serotypes also bind with an unknown protein having the property of serotype specific binding (Figure1) [13]. After initial attachment of the virus with particular receptors on the surface of host cell, the viral particle is fused into acidic lysosomes through receptor-mediated endocytosis. After that, viral particle is uncoated and the RNA is released in host cell where it directs the synthesis of viral proteins (Figure2). Once all the essential proteins are synthesized, viral RNA starts copying to generate a minus strand, which is then transcribed to new plus stranded molecules. In only few hours after infection, tens of thousands copies of viral molecules are produced from a single viral molecule leading to cell damage and in severe cases to death. Viral-encoded RNA-dependent RNA polymerases (RdRps) and other cellular factors are responsible for catalyzing the infection cycle of dengue virus [14]. The mechanism of vascular permeability and haemorrhaging is not clearly known. To understand these mechanism studies are being focused on the role of T-cell immune response. After 1–2 days of onset fever during secondary infection, high concentrations of interferon alpha were recorded [15]. High concentrations of soluble interleukin 2 receptor, soluble CD4, soluble CD8, interleukin 2, and interferon γ were also studied during the onset of vascular permeability [16].

## DENV genome

**Figure 3:**
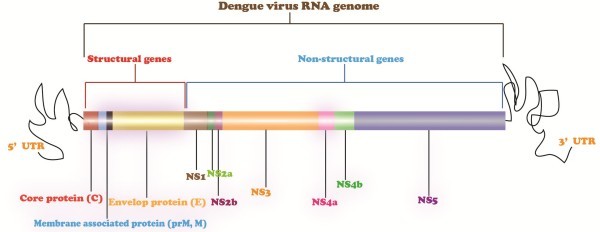
**Dengue virus genome.** Dengue virus genome encodes 10 viral proteins including 3 structural proteins (C, M, and E) responsible for viral structure and viral attachment to host cell and 7 non-structural proteins (NS1, NS2A, NS2B, NS3, NS4A, NS4B, NS5) that are involved in viral replication and other cellular function.

Dengue virus is plus stranded RNA virus with genome of 11 kilobases. The dengue virus genome with a large open reading frame encodes a polyprotein precursor of about 3000 amino acids that are processed cotranslationally and posttranslationally by viral and host proteases. This polyprotein precursor is cleaved to generate at least 10 proteins like other viruses belonging to Flaviviridae family [17]. These proteins include three structural proteins, nucleocapsid or core protein (C), a membrane associated protein (M), an envelope protein (E) and seven nonstructural proteins. The order of genes is in 5_-CprM (M)-E-NS1-NS2A-NS2B-NS3-NS4A-NS4B-NS5-3 (Figure3) [18]. These viral proteins are responsible for viral replication and various cellular functions [19]. Viruses upon infection activates interferon (IFN) signaling pathway but dengue develops interferon resistance. NS2a, NS4a and NS4b block the interferon cascade to escape the immune response. NS4b inhibits interferon cascade by blocking STAT-1 phosphorylation [17].

## DENV proteins and therapeutic targets

### DENV structural proteins

DENV has three structural proteins: Core protein (C), a membrane associated protein (M) and Envelop protein (E). These proteins are involved in defining the structure of the virus.

#### DENV core protein (C)

DENV core protein is a highly basic protein responsible for assembly of nucleocapsid through interaction with RNA but only a little is known about C protein. Structure of the protein revealed that the C protein dimer had high net charge and there is asymmetric distribution of basic residues over the surface of protein [20]. Anti DEN-2 core protein monoclonal antibodies (MAbs) reacted with antigens in cytoplasm and in the nucleus of DEN-2 and DEN-4 but not DEN-1 and DEN-3. These MAbs also reacted with core proteins of DEN-1, DEN-2 and DEN-4 in western blot studies. All MAbs react with region (9RNTPFNMLKRE19) of DEN-2 core protein revealed by PEPSCAN epitope mapping [21]. The core protein C localizes in nucleus. It was determined that a motif (RKeigrmlnilnRRRR, located at aa 85–100) is responsible for the nuclear localization of the core protein and without this motif core protein resides in the cytoplasm of DEN-infected cells [22].

#### Membrane associated protein (M)

Membrane associated protein is a membrane glycoprotein works as a part of nucleocapsid and assists to envelop protein to form mature virions. Antibodies against prM protein were developed for Japanese encephalitis virus (JEV), West Nile virus (WNV) and dengue virus. Antibodies against the prM protein of JEV do not react with the prM of dengue viruses or West Nile virus. prM protein can be used to investigate differentiating antibody responses to difference flaviviruses [23]. C-prM was first identified by immuno-precipitating the Aedes albopictus cells infected with DEN-2. These cells were cleaved to produce membrane associated protein (M) and non membrane fragment (pr). This cleavage was less efficient in mosquito cells. Proteins containing fragment was then fused with staphylococcal protein A. The fused protein was stable and were used to generate antisera in rabbits [24]. The E-prM interactions in dengue virus are mediated by domains in the carboxy-terminal anchoring domain of E, while cell activity is mediated by trypsin-releasable ectodomain of E protein [25].

#### Envelop protein (E)

Envelop protein is present on the surface of the virus and is extremely involved in virus attachment with host cell through cell receptors like heparin sulphate DC-SIGN. It is most important protein for the entry of virus in cell. This protein has three domains, domain I (structural domain), domain II (dimmers) and domain III (binding domain). Dimmer domain links structural and binding domain [26,27]. The Dengue virus enters a host cell when viral envelop protein binds to receptor and responds by conformational rearrangement to the reduced pH of an endosome. The conformational change induces fusion of viral and host-cell membranes [28]. Flaviviruses uses a fusion mechanism by inserting distal β barrels of domain II of E protein into cellular membrane [29]. Crystal structure of soluble ectodermal domain of E revealed a hydrophobic pocket lined by residues that influence the pH for fusion. This pocket opens and closes in the β-hairpin at the interface between two domains. Thus, it can be used to test antiviral compounds [30].

### DENV non-structural proteins

DENV genome encodes seven non-structural proteins, including NS1, NS2a, NS2b, NS3, NS4a, NS4b and NS5. These proteins are responsible for viral replication and other cellular functions.

### NS1 protein

NS1 is expressed on the surface of the infected cell and can serve as an antibody target for dengue infection [31]. NS1 protein is involved in RNA replication as it resides to site of RNA replication but still the role of NS1 is unclear. NS1 Antigen was detected from the 1^st^ day to day 9 of infection. This was even detected when viral RNA was negative in RT-PCR. Its level differed in individuals ranging from a couple of nanograms per milliliter to several micrograms per milliliter. Thus, detection of NS1 protein may allow diagnosis of dengue infection in early stages [32]. In a study, plasma level of secreted NS1 (sNS1) were measured in 32 children infected with DEN-2. sNS1 level was higher in patients with DHF than DF. Within, 72 hours of infection, sNS1 level was ≥600 ng/mL indicating the risk of developing DHF [33]. NS1 mediated complement activation leads to generation of SC5b-9, may be involved in vascular leakage in DHF/DSS patients [34].

#### NS2A protein

NS2A protein is majorly involved in RNA replication and viral assembly. A mutation in NS2A protein blocked virus replication. NS2A also inhibits the IFN-β promoter-driven transcription, thus serves as interferon (IFN) antagonist [17,35]. A single amino acid mutation in NS2A was identified that reduces inhibitory activity of NS2 [35] . NS2A may be a cis-acting protease that cleaves itself from NS1 protein or may provide recognition sequences to cellular proteases that cleaves their junction [36]. NS2A were found to be located in discrete foci in the cytoplasm at 16 and 24 hours pos-infection [37]. Using KUN replicon vectors for gene expression NS2 has been identified as new target for developing live flavivirus vaccines [35].

#### NS2B protein

NS2B works as a cofactor in NS2B-NS3 serine protease. NS2B is required for the cleavage of NS2A/NS2B and NS2B/NS3.NS2B is also required for NS3/NS4A cleavage and for other internal cleavages [38].Wang and colleagues investigated the extent of intra host sequence variation of NS2B, derived from plasma dengue viruses from 18 DEN-3 infected patients. They demonstrated the quasi species structure of dengue virus in-vivo and suggested that sequence variation in DEN-3 NS2B was likely to reflect genetic drift [39]. Falgout and colleagues determined a 40 amino acid segment of NS2B (DEN4 amino acids 1396 to 1435) essential for protease activity. This segment constitutes a hydrophilic domain surrounded by hydrophobic regions. The hydrophilic domain of NS2B will prove to be essential for protease activity [40].

#### NS3 protein

NS3 of DEN-2 is second largest non-structural protein having serine protease at N-terminus and NTPase helicase RNA triphosphate (RTPase) at C-terminus end. [41]. NS3 is also involved in RNA replication and helps in regulating poly protein processing [42,43]. Teo and colleagues proposed that NS3 results from cleavage at site …R457R / GR460… in RNA helicase sequence motif, and it does not require prior cleavage of NS2B/NS3 [43]. NS3 was immune-dominant in CD4+ T-cell response of DEN-3 infected donor [44]. Immunoblotting and coimmunoprecipitation studies showed that NS3 protein also interacts with NS5 protein in DEN-2 infected monkey kidney cells and HeLa cells co-infected with a recombinant vaccinia virus [45].Chow and colleagues studied NS3 gene targets of all four dengue serotypes using RT-PCR followed by ELISA. The results were high spectrophotometric absorbance obtained by hybridization consensus amplification products with their probe [46]. Non substrate based inhibitors have potency to bind in the P1 subpocket of catalytic site of DENV NS3 protease [47]. Recently, the release of 3D structure of NS2B, NS3 of DEN-2 has opened new ways to discover potent antiviral compounds that can target all four dengue serotypes [48]. Due to recent availability of NS3 helicase 3D structure, it was discovered that RTPase uses helicase scaffold to perform their activity [49]. NS3 because of its domains and their role in viral replication are considered to be an important drug target against dengue virus infection. But still there is a need to develop antiviral compounds that can target all four serotypes of dengue with same efficiency. Evaluation work of NS3 domains from all four serotypes is currently in progress.

#### NS4A protein

NS4A is the least characterized protein. At its C-terminus end, it is highly hydrophilic and serves as a signal to translocate NS4B to the endoplasmic reticulum lumen. In membrane bound viral replication complex (RC), NS4A forms components that help in maintaining this complex [50]. NS4A has a role in replication cycle, membrane rearrangements and their regulation. The N-terminus of NS4A is generated by cleavage by viral proteinase NS3 in a cell-free system [51]. NS4A is located in discrete foci in the cytoplasm of infected cell at both 16 and 24 hours of post-infection, partially coincident with dsRNA foci [37].

#### NS4B protein

NS4B interacts with NS3 to regulate RNA replication. When treated with IFN-β or IFN-γ, NS4B expressing cells did not exhibit signal transducer and activator of transcription (STAT 1), thus indicated that NS4B may be involved in blocking IFN signaling and can be implicated as IFN-signaling inhibitor [17]. Function of NS4B is conserved in West Nile and yellow fever viruses. First, 125 amino acids of dengue virus NS4B is sufficient for inhibition of alpha/beta IFN (IFN-α/β) signaling [52].

#### NS5 protein

NS5 has a methyltransferase (MTase) domain at N-terminus end and a polymerase domain at C-terminus end. NS5 has a role in RNA replication due to presence of RNA dependent RNA polymerase activity. It is also involved in de novo initiated RNA synthesis [53].Charge-to-alanine mutagenesis of DEN-4 NS5 gene generated a collection of attenuating mutations for potential use in a recombinant live attenuated DEN vaccine [54]. NS5 in the hyper-phosphorylated form does not interact with NS3 due to action of functional nuclear localization sequence within the inter domain region of NS5 (residues 369–405). Importin-β with NS5 has implication for the mechanism by which this normally cytoplasmic protein may be targeted to the nucleus [55]. DENV NS5MTase structure with the co-product methyl transfer S-adenosyl-L-homocystein is a intial step for developing antiviral drugs against DENV [56]. C-terminal domain of NS5 possesses RNA-dependent RNA polymerase (RdRp) activity that is involved in viral replication and is an important drug target for discovering new drugs [53].

## Prevalence in pakistan

Dengue has a worldwide history of about 200–400 years, mostly causing infection in tropical and sub-tropical areas. Dengue virus is believed to come in Pakistan with tyres at Karachi sea port carrying eggs of infected mosquitoes. To date, dengue virus infection has caused several outbreaks in Pakistan [57,58]. Until 1994, there was no valid data available about dengue infection cases in Pakistan. In 1982, Dengue was identified in Pakistan, Punjab. Out of 174 patients, 12 were dengue virus positive [58]. In 1985, a research was conducted to study the prevalence of dengue virus infection in Pakistan. It showed that about 50-60% of the Pakistanis, especially those living in Karachi were haem agglutination inhibition (HI) antibody positive for West Nile, Japanese encephalitis and DENV-2 Flaviviruses. These cases rapidly increased from July to October in patients ranging from 6 to 20 year age [59]. In 1994, first outbreak of DHF was reported in Pakistan [60]. Out of 16 patients, 15 had dengue IgM identified using DENV2 antigen. It was also observed that in three out of ten patients of dengue virus were infected with DEN-1 and DEN-2 [61]. In 1995, DEN-2 infection was reported from Balochistan [62,63]. In 1998, DEN-1 and DEN-2 were found in patients using ELISA study [64]. In 2005, outbreak of DHF in Karachi, DEN-3 was reported among the few tested patients. In serum of children in karachi, DEN-1 and DEN-2 were found using serological studies [64,65]. DEN-2 and DEN-3 were found to be co-circulated during 2006 outbreak in Karachi [57,66]. DEN-3 in 2006 outbreak in Pakistan was found to be closely related to DEN-3 in 2004 outbreak in New Delhi [67]. In 2008, a dengue outbreak was reported in Lahore infecting a large number of citizens of Lahore. Samples were found to have DEN-4, DEN-2 and DEN-3 infection [63]. In 2009, it was reported that children living in Karachi had high levels of anti-dengue IdM antibody [68]. Samples had concurrent infection with serotypes DEN-2 and DEN-3. Studies showed that serotype DEN-2 was dominant in samples of dengue virus infection collected during the period of three years (2007–2009) [7]. In November 2010, it was reported by a private news channel that Out of the 5,050 patients, 2,350 patients were from Sindh, 1,885 from Punjab and at 158 patients from Khyber Pakhtunkhwa [69].The samples had an infection with DEN-2 and DEN-1 (Table1) [70].

**Table 1:** Year-wise prevalence of dengue virus serotypes in Pakistan

**Year**	**DENV Serotype**	**Reference**
**1994**	DEN-2	[61]
**1995**	DEN-2	[62,63]
**1998**	DEN-1, DEN-2	[64]
**2005**	DEN-1, DEN-2, DEN-3	[64,65]
**2006**	DEN-2, DEN-3	[57,66]
**2008**	DEN-2, DEN-3, DEN-4	[63]
**2009**	DEN-2, DEN-3	[7]
**2010**	DEN-1, DEN-2	[70]

During 2011, the disease rapidly assumed the proportions of epidemic, specifically in Punjab and particularly Lahore where, in September, more than 250 people were reported dead and, according to the Punjab Health Department, over 12,000 people were infected during January to September 2011. Sindh, too, was hit by the dengue virus. According to the Sindh Provincial Dengue Surveillance Cell, nearly 400 people were infected with the virus in few months with over 300 people in Karachi alone, and the death toll stood at five at the end of September 2011. In February 2012, 73 confirmed cases of dengue in Lahore and 13 other regions of the state of Punjab were reported (http://www.paperpk.com/news/index.php/new-dengue-cases-punjab-2012/). The year 2007 and 2011 has been worst years in regard of dengue virus infection in Pakistan. Dengue virus circulates in Pakistan throughout the year with a peak incidence in the post monsoon period. Recent flood in Pakistan made the situation worse [12].

## Diagnosis

Dengue virus can cause mild fever to Dengue fever (DF) or more severely DHF/DSS. DF causes fever in 3–5 days of infection, in which patient can feel headache, joint pain and rashes on the body with thrombocytopenia and relative leucopenia. Both DHF and DSS have same symptoms as of DF but they also have increased vascular permeability and hemorrhage, can lead to death. Dengue infection is mostly clinically diagnosed through symptoms. However, diagnosing dengue infection through symptoms is not reliable, and it needs laboratory studies to confirm the presence of dengue virus [71,72]. Differential diagnosis is also important because dengue fever can easily be confused with non-dengue illnesses, particularly in nonepidemic situations. Depending on the geographical origin of the patient, other etiologies, including non-dengue flavivirus infections should be ruled out.

Laboratory diagnosis of dengue virus infection can be made by the detection of particular virus, viral antigen, genomic sequence, and/or antibodies. IgM captured ELISA, virus isolation in mosquito cell lines and live mosquitoes, dengue specific monoclonal antibodies and detection of viral RNA by nucleic acid amplification tests (NAAT) have all represented major advances in dengue diagnosis. There are two stages of diagnosing dengue infection. In stage 1, fever is accompanied by NS1 antigens in blood and in the stage 2, post-febrile period in which IgM and IgG antibodies are in excess. In acute phase of dengue, patients generally suffer from flu-like fever and seek medical attention in first two days. During this phase, diagnosis is possible by detecting viral RNA/proteins in patient blood. If after two days fever is not recovered, then patient should be advised for a complete blood checkup (CBC). After the checkup, if platelet count is below normal range (150,000 to 450,000 platelets per microliter of blood), and WBC count is below normal range (4300–10,800 WBCs per cubic millimeter (cmm) of blood) then patient should go for dengue antigen test. Patients with DHF/DSS should go for serological test. Using serotype specific NS3 primers, infection was identified in 80% patients [73]. Dengue viral RNA can be detected in early phase of infection using real-time reverse transcriptase (RT) PCR. These techniques are reliable in detecting dengue virus but are costly [74,75]. ELISA test using dengue-specific NS1 monoclonal antibody is being used to detect NS1 in blood of the patients and to characterize the primary and secondary infection in patients [76,77]. MAC-ELISA assays when combined with NS1 Ag holds promise to detect the dengue viruses in early phases of infection [78]. Currently used laboratory methods are: capture ELISAs, immunofluorescence tests, and hemagglutination assays [79]. A recent study was conducted to use saliva to detect the dengue virus specific immunoglobulin A (IgA) in early phase of dengue infection. This technique can be very helpful in dengue endemic regions, where the majority of dengue cases are secondary [80]. A new diagnostic test has recently been approved by US Food and Drug administration, the CDC DENV-1-4 Real time RT PCR Assay developed by Centers for Disease Control and Prevention to detect the dengue viruses in infected patients [81]. However, there is a need to develop an effective and low cost diagnostic methods that can detect the presence of dengue virus in acute phase of infection so that clinical preventive measure can be taken to avoid any severity of the disease.

## Prevention & treatment

To date, there is no specific treatment available for dengue virus infection. There are three ways to solve the problem of dengue infection. The First is to use a preventive measure by avoiding contact with infected mosquitoes. Aedes mosquitoes bite during daytime and its contact can be avoided by: properly managing waste and improving storage of water, removing all sources of stagnant water, using household pesticides to kill mosquitoes, using mosquito coils and nets, wearing long-sleeved shirts, socks and trousers and using insect repellent to avoid mosquitoes. Insecticide treated nets (ITNs) are available to protect young children, pregnant women, old people, in addition to others who may rest during the day [12]. Government of Pakistan and Punjab, Pakistan is working on preventive measure by increasing awareness of dengue among people. Spraying teams for purpose of fumigation and spraying are organized to kill Aedes mosquito known to infect people (http://en.wikipedia.org/wiki/2011_dengue_outbreak_in_Pakistan). The Second is vaccination, which is currently not available and the Third is drug therapy but no antiviral drugs are available to target dengue virus [82]. However, supportive care and treatment can save a patient infected with dengue fever. Fever can be treated by anti-pyretics, like paracetamol. Joint pain can be treated by analgesics or painkiller tablets. In case of DHF/DSS, patients must be hospitalized. Dehydration can be prevented by oral rehydration therapy and if oral intake is imposible then intravenous fluid replacement can be used to prevent shock in infected patients. If platelet level drops below 20,000 or if there is significant bleeding, then platelet transfusion is recommended. Drugs such as aspirin, brufen and non-steroidal anti- inflammatory should be avoided as they may worsen the bleeding tendency. Any medicine that decreases the platelet level should be avoided (http://www.denguevirusnet.com/treatment.html).

## Medicinal plants as dengue virus inhibitors

Plants have been traditionally used to cure a number of human diseases. To date, few plants derivatives due to their medicinal properties have successfully been tested against viral diseases. The first step of DENV cycle is to attach with host via host receptors. Envelop protein (E) is involved in viral and host attachment. Thus, dengue infection can be inhibited by targeting envelop protein/inhibiting host-viral interactions. NS2-NS3 protease and NS5 also serve as important antiviral drug targets due to their role in viral replication and other cellular processes. To date, many medicinal plants have been tested against DENV and some of them showed significant inhibition effects in the DENV replication cycle.

Antiviral effects of methanolic extracts of Andrographis paniculata, Citrus limon, Cymbopogan citrates, Momordica charantia, Ocimum sanctum and Pelargonium citrosum on dengue virus serotype 1 (DENV-1) were investigated by Tang and colleagues. A. paniculata showed the most antiviral inhibitory effect followed by M. chrantia in in-vitro assays. Both these plants can be advantageous in developing novel antiviral compounds [83]. Carica papaya contains two important biologically active compounds, chymopapain and papain, which are used in digestive disorders [84]. C. papaya leaves extract prepared in water has been tested against dengue fever. After the administration of aqueous extract in dengue infected patient, the platelet count increased from 55x10^3^/μL to 168x10^3^/μL, White blood cells from 3.7x10^3^/μL to 7.7x10^3^/μL and neutrophils from 46% to 78%. Thus carica papya can be used to target dengue fever [85]. Extracts of neem leaves and pure neem compound (Azadirachtin) were tested against DENV-2 replication. In-vitro activity was assessed in C_6/36_ (cloned cells of larvae of *Aedesalbopictus*) cells. Aqueous neem extracts at its maximum nontoxic concentration of 1.897 mg/ml, completely inhibited 100–10,000 TCID_50_ of virus as indicated by the absence of cytopathic effects [86]. Talarico and colleagues investigated lambda- and iota-carrageenans, sulfated polysaccharides containing linear chains of galactopyranosyl residues against DEN-2, DEN-3 using Vero and HepG2 cells and found that these compounds are potent dengue inhibitors. The inhibitory action was exerted by dual interference with virus adsorption and internalization of nucleocapsid into the cytoplasm. Carrageenans did not interfere with viral protein synthesis and virus multiplication. Thus, carrageenans can be used in developing new therapies by interfering with virus adsorption in host cell [87]. Rehman and colleagues investigated Quercus lusitanica extract against DEN-2 replication. At a concentration of 180 μg/ml, Q. lusitanica was found to completely inhibit dengue virus infection. Furthermore, methyl gallate from fractionalized crude extracts showed 96% inhibition at the maximum non-toxic dose (MNTD) of 100 μg/mL [88]. DEN-2 infection was inhibited by WSS45 (Sulfate derivative of an alpha-D-glucan) derived from Gastrodia B1 in BHK (Baby hamster kidney fibroblast cells) cells with an EC (50) value of 0.68+/−0.17 μg/mL. WSS45 interfered with virus adsorption but showed no viricidal effect. Thus, WSS45 can be used to increase virus detaching from the host cell surface [89]. Replication of DEN-2 was significantly reduced by two compounds; 1-beta-d-ribofuranosyl-3-ethynyl-[1, 2, 4]triazole (ETAR) and 1-beta-d-ribofuranosyl-4-ethynyl-[1, 3]imidazole (IM18). ETAR also reduced replication of DEN-1, DEN-3 and DEN-4. Therefore, ETAR can be used as a potential therapeutic drug against dengue viruses [90]. Kiat and colleagues investigated two cyclohexenyl chalcone derivatives of Boesenbergia rotunda (L.), 4-hydroxypanduratin A and panduratin A and showed inhibitory activity of both compouds against DEN-2 NS3 protease with the Ki values of 21 and 25 μM, respectively [91]. A series of new mono- and dialkylated flavanones having NS5 RNA-dependent RNA polymerase (RdRp) inhibiting activity named chartaceones A-F (1–6) along with pinocembrin was identified from Crypotocarya chartacea.Chartaceones C-F (3–6) which are dialkylated flavanones showed significant NS5 RdRp inhibiting activity, with IC(50) ranging from 1.8 to 4.2 μM [92]. Zandi and colleagues investigated four bioflavanoids (quercetin, daidzein, naringin and hesperetin) against DEN-2 using Vero cells and proved that only quercetin had significant anti-DEN-2 inhibitory actitivty. Thus, there is a need to further investigate these compounds to develop novel inhibitors against DENV [93]. Three flavonoids; fisetin, naringenin and rutin were tested against DENV-2 serotype using foci forming unit reduction assay (FFURA) and quantitative real-time polymerase chain amplification (qRT-PCR). After viral adsorption, Fisetin was added that lead to DENV-2 replication inhibition with a half maximal inhibition concentration (IC_50_) value of 55 μg/mL and selectivity index (SI) of 4.49. In Vero cells, rutin and naringenin did not inhibit DENV-2 replication [94]. Recently, Tang and colleagues investigated methanolic extracts of Andrographis paniculata, Citrus limon, Cymbopogon citratus, Momordica charantia, Ocimum sanctum and Pelargonium citrosum on DEN-1. Among all the six medicinal plants, A. paniculata showed the most antiviral inhibitory effects followed by M. charantia. Thus, these two plants need further investigations to develop potential dengue treatment [83].

## Conclusion

Dengue infection has emerged as a major health concern in Southeast Asia, the pacific and America. Dengue virus has become a serious issue in Pakistan as it has caused many endemics starting from 1994 to 2011. Developing tetravalent vaccine against all four dengue serotypes is quite challenging. To date, there is no licensed vaccine available for dengue virus. Therefore, there is an urgent need to develop an alternative solution to combat this endemic infection. Currently, there are no antiviral compounds available against dengue virus and there is a need to develop antiviral compounds that can target all four serotypes of dengue with same efficiency. Dengue virus enveloped protein is involved in virus cell entry; NS3 and NS5 are involved in viral replication and other cellular processes; therefore, these can serve as an important drug target to combat this life-threatening disease. Several medicinal plants have been tested against dengue virus entry and replication; many of them showed significant inhibitory effects. Nevertheless, it will be very exciting to see these medicinal plants as potential DENV inhibitors to progress through clinical developments and, hopefully, provide dengue patients with much needed, more effective therapies.

## Competing interests

All authors have no kind of institutional and financial competing interests.

## Author contributions

UAA designed the study and SI wrote the manuscript. Both the authors read and approved the final manuscript.

## Authors’ information

Sobia Idrees (MPhil student), Usman A Ashfaq (PhD molecular Biology and Group leader, Human Molecular Biology Group, Department of Bioinformatics and Biotechnology, GCU, Faisalabad.
